# 386. *Clostridioides difficile* PCR Cycle Threshold to Determine Toxin Positivity Status

**DOI:** 10.1093/ofid/ofac492.464

**Published:** 2022-12-15

**Authors:** Andrew S Crone, Andrew M Skinner, Lorinda M Wright, Adam K Cheknis, Stuart Johnson, Susan M Pacheco

**Affiliations:** Loyola University Medical Center, Edward Hines Jr. VA Medical Center, Chicago, Illinois; Loyola University Chicago Stritch School of Medicine, Maywood, Illinois; Edward Hines VA Hospital, Hines, Illinois; Edward Hines Jr. VA Hospital, Hines, Illinois; Hines VA Hospital and Loyola University Medical Center, Hines, Illinois; Edward Hines, Jr. VA Hospital and Loyola University Medical Center, Hines, Illinois

## Abstract

**Background:**

Many clinical laboratories rely on polymerase chain reaction (PCR) as the sole test for diagnosis of *C. difficile* infection (CDI). The objective of this study was to determine if the *C. difficile* (CD) PCR cycle threshold could be used to predict a positive CD toxin test.

**Methods:**

We reviewed the characteristics and outcomes of 149 consecutive patients who tested positive for by PCR (Xpert CD, Cepheid) between October 2019 and December 2021 at one VA Hospital where reflex toxin testing (Cdiff quick check complete, Alere/TechLab) was performed after a positive PCR result. Baseline characteristics, symptoms, initial laboratory data, and treatments were compared as well as clinical outcomes. Determination of CDI or CD colonization was made for each patient after review of symptoms: including chronicity of symptoms, laboratory values, stool frequency, use of laxatives, and response to treatment. The cycle threshold for the CD-PCR results were recorded and positive stools were cultured for CD.

**Results:**

Toxin testing was positive in 38% (57/149) of cases. The mean PCR cycle threshold value for all specimens tested was 27.89 (95% CI [27.14, 28.66]). Toxin-positive stools had lower mean PCR cycle thresholds when compared to toxin-negative stools (24.56 and 29.97, respectively p < 0.0001). Among the 149 cases reviewed, 109 were determined to be CDI (73%), 36 were colonized (24%), and 4 cases were undetermined. Among those with toxin-positive stools, 96% (54/56) patients were determined to have CDI. 98% (56/57) of all toxin-positive stools had a cycle threshold of ≤ 32.2 compared to 63% (58/92) of toxin-negative stools, resulting in a negative predictive value of 97.2% (p < 0.0001). The mean cycle threshold was lower in patients who were determined to have a CDI when compared to colonized patients (27.02 vs 30.44, p = 0.0001). [Figure] Of the patients that had CDI, 83% (91/109) had a cycle threshold of ≤ 32.2 and 58% (21/36) of colonized patients had a cycle threshold of ≤ 32.2.

**Figure:**
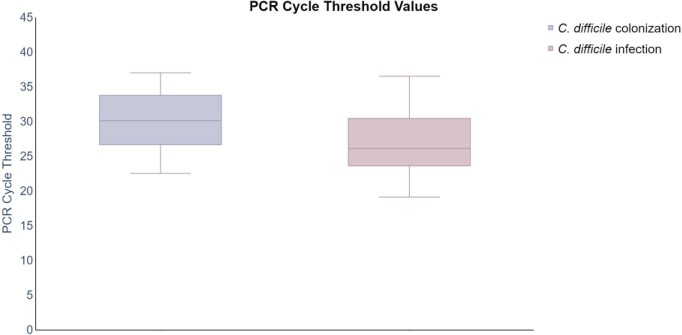

**Conclusion:**

Conclusions: In a setting where a CD toxin assay is not readily available, a CD stool PCR cycle threshold could be used to predict toxin status. In our study, a cycle threshold of ≤ 32.2 predicted both toxin-positivity and CDI.

**Disclosures:**

**Stuart Johnson, M.D.**, Ferring Pharmaceuticals: Membership on Ferring Publication Steering Committee|Ferring Pharmaceuticals: Employee|Summit Plc: Advisor/Consultant.

